# Biomimetic Robotics and Sensing for Healthcare Applications and Rehabilitation: A Systematic Review

**DOI:** 10.3390/biomimetics10070466

**Published:** 2025-07-16

**Authors:** H. M. K. K. M. B. Herath, Nuwan Madusanka, S. L. P. Yasakethu, Chaminda Hewage, Byeong-Il Lee

**Affiliations:** 1 Industry 4.0 Convergence Bionics Engineering, Pukyoung National University, Busan 48513, Republic of Korea; kasunkh@pukyong.ac.kr; 2 Digital Healthcare Research Center, Pukyong National University, Busan 48513, Republic of Korea; nuwanv@pknu.ac.kr; 3 Faculty of Engineering, Sri Lanka Technology Campus, Padukka 10500, Sri Lanka; lasithy@sltc.ac.lk; 4 Cardiff School of Technologies, Cardiff Metropolitan University, Cardiff CF23 6PS, UK; chewage@cardiffmet.ac.uk; 5 Division of Smart Healthcare, College of Information Technology and Convergence, Pukyong National University, Busan 48513, Republic of Korea

**Keywords:** bio-inspired robotics, medical diagnostics, rehabilitation technologies, surgical innovations, biomimetic sensing

## Abstract

Biomimetic robotics and sensor technologies are reshaping the landscape of healthcare and rehabilitation. Despite significant progress across various domains, many areas within healthcare still demand further bio-inspired innovations. To advance this field effectively, it is essential to synthesize existing research, identify persistent knowledge gaps, and establish clear frameworks to guide future developments. This systematic review addresses these needs by analyzing 89 peer-reviewed sources retrieved from the Scopus database, focusing on the application of biomimetic robotics and sensing technologies in healthcare and rehabilitation contexts. The findings indicate a predominant focus on enhancing human mobility and support, with rehabilitative and assistive technologies comprising 61.8% of the reviewed literature. Additionally, 12.36% of the studies incorporate intelligent control systems and Artificial Intelligence (AI), reflecting a growing trend toward adaptive and autonomous solutions. Further technological advancements are demonstrated by research in bioengineering applications (13.48%) and innovations in soft robotics with smart actuation mechanisms (11.24%). The development of medical robots (7.87%) and wearable robotics, including exosuits (10.11%), underscores specific progress in clinical and patient-centered care. Moreover, the emergence of transdisciplinary approaches, present in 6.74% of the studies, highlights the increasing convergence of diverse fields in tackling complex healthcare challenges. By consolidating current research efforts, this review aims to provide a comprehensive overview of the state of the art, serving as a foundation for future investigations aimed at improving healthcare outcomes and enhancing quality of life.

## 1. Introduction

Inspired by biological systems’ exceptional functionality and adaptability, biomimetic robotics and sensing technologies have become revolutionary tools in the healthcare and rehabilitation fields [1]. These cutting-edge technologies aim to mimic the complex workings of nature, utilizing the complementary fields of biology and engineering to improve patient care and rehabilitation results. Biomimetic sensors and robots provide novel answers to long-standing problems in medical and rehabilitation procedures by mimicking the shape and functionality of biological species, from the agility of animal locomotion to the dexterity of the human hand [2,3]. In addition to providing more individualized and effective care routes, the smooth integration of these technologies into healthcare settings can transform diagnosis, treatment, and monitoring. From bio-inspired materials to sensors and assistive medical systems, the healthcare sector is actively harnessing biomimetics.

The capacity to mimic natural movements and responses, which are frequently more flexible and effective than those of strictly constructed systems, drives biomimetic robotics and sensing [4,5]. These features, such as rehabilitation robots, exoskeletons, and prosthetics, are essential for applications that restore or enhance human function. These systems enable real-time, adaptive interactions between patients and assistive devices, which are made possible by sensing technologies inspired by biological processes, such as tactile sensors that simulate the skin’s sensitivity or visual systems that resemble the human eye [6,7]. More dynamic and successful rehabilitation techniques are made possible by these bio-inspired sensors’ ability to track physiological parameters, identify minute changes in a patient’s state, and modify treatment approaches accordingly [8,9].

**Figure 1:**
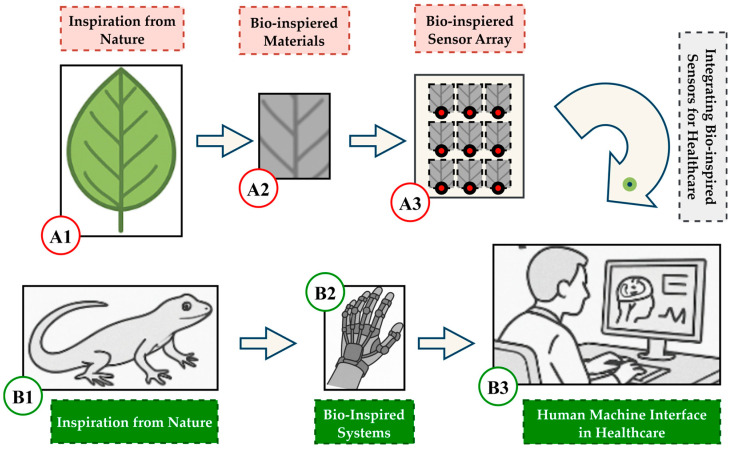
Conceptual pathways from natural inspiration (e.g., leaves and lizards) to biomimetic healthcare devices like sensor arrays and exosuits.

Figure 1 presents two conceptual pathways showing how bio-inspired ideas lead to technological innovations. Path A traces inspiration from natural structures (like a leaf) to bio-inspired materials and sensor arrays. The Path B follows animal-inspired systems (like a lizard) to robotic devices and healthcare interfaces, such as exoskeletons. As shown in the illustration, both contributed to the role of nature-inspired design in advancing sensing technologies and human–machine interfaces in the healthcare sector.

Despite notable advancements in the multidisciplinary field of biomimetic robotics and sensing technologies, particularly within healthcare and rehabilitation contexts, a comprehensive understanding of the field’s current state remains insufficient. Systematic reviews are crucial in synthesizing existing research, identifying knowledge gaps, and establishing coherent frameworks to guide future innovations. This systematic review offers a detailed examination of biomimetic robotics and sensing technologies applied in healthcare and rehabilitation, focusing on their design principles, functional mechanisms, clinical efficacy, and potential for integration across diverse medical settings.

By evaluating the current landscape and highlighting promising directions, this review underscores both the technological progress achieved and the essential role of interdisciplinary collaboration in shaping the future of patient-centered care. The exploration of biomimetic robotics and sensing technologies is fundamentally driven by a commitment to enhancing quality of life, restoring lost functions, and expanding the capabilities of both patients and healthcare providers.

What distinguishes this publication from existing reviews is its systematic and up-to-date synthesis of 89 studies published between 2020 and early 2025. The review employs a PRISMA-based methodology to identify relevant studies and categorizes the findings into interdisciplinary technological clusters. In addition to documenting recent technological advancements, the review identifies critical areas for future inquiry, such as the development of compliant actuators and the challenges of clinical translation, offering valuable insights to inform subsequent research and innovation.

## 2. Source Screening Methodology

**Figure 2:**
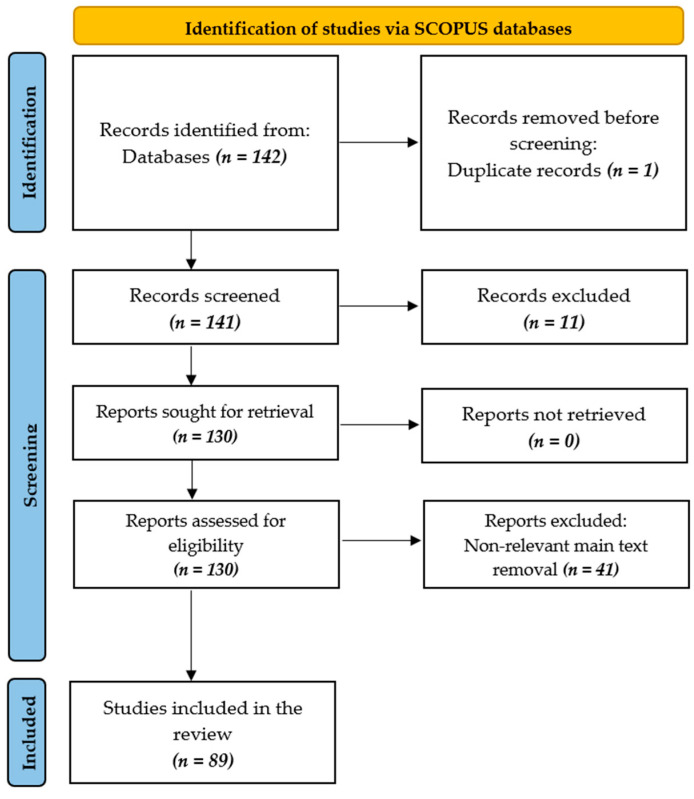
PRISMA model of the review procedure.

This section outlines the review design methodology. The authors conducted the literature screening process by the Preferred Reporting Items for Systematic Reviews and Meta-Analyses (PRISMA) guidelines [10]. Figure 2 displays the PRISMA flow diagram that depicts the review procedure.

The PRISMA checklist was also used to ensure transparent and standardized reporting. The Scopus database was selected due to its extensive coverage of peer-reviewed engineering and medical publications. Under the PRISMA guidelines, the initial literature search was conducted using the Scopus database, targeting publications from 2020 to 2025 with keywords and keyword combinations such as “bio-inspired,” “biomimetic,” “biomimicry,” “rehabilitation,” “diagnosis,” “surgical technique,” “robotics,” and “sensing.” The search was restricted to English-language documents, including articles, conference papers, and book chapters. This yielded 142 records, from which studies unrelated to direct medical, diagnostic, and rehabilitative applications and duplicated records were excluded, resulting in 130 sources for further analysis. The 41 more sources were omitted from a content analysis because they were irrelevant (purely mechanical or structural biomimetic systems not directly used in rehabilitation, diagnosis, surgery, or sensing). Eighty-nine sources were selected for the final review, and this review study is based on the information derived from those sources.

**Figure 3:**
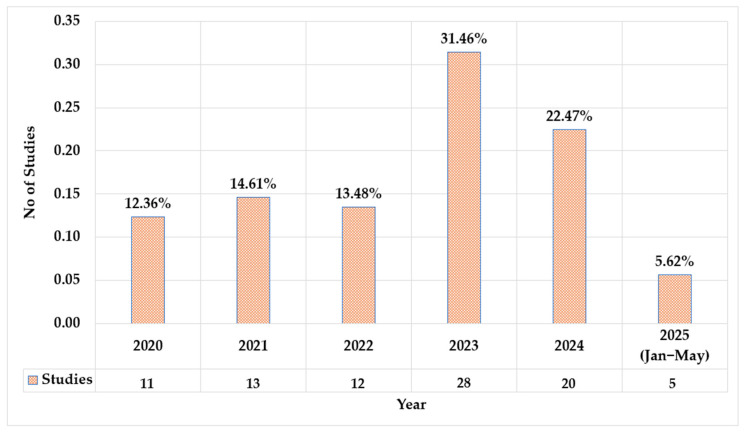
Distribution of the selected studies for the review.

Figure 3 illustrates the annual distribution of studies from 2020 to May 2025. The number of studies remained relatively stable between 2020, 2021, and 2022, ranging from 11, 13, and 12, with corresponding percentages between 12.36%, 14.61%, and 13.48%. A significant increase occurred in 2023, reaching the highest number of studies at 28 (31.46%). This was followed by a slight decrease in 2024, with 20 studies (22.47%). For the partial year of 2025 (January to May), the figure dropped considerably to 5 studies, accounting for only 5.62%. This trend also emphasizes that there is still a demand for development and the filling of research gaps in the domain.

**Figure 4:**
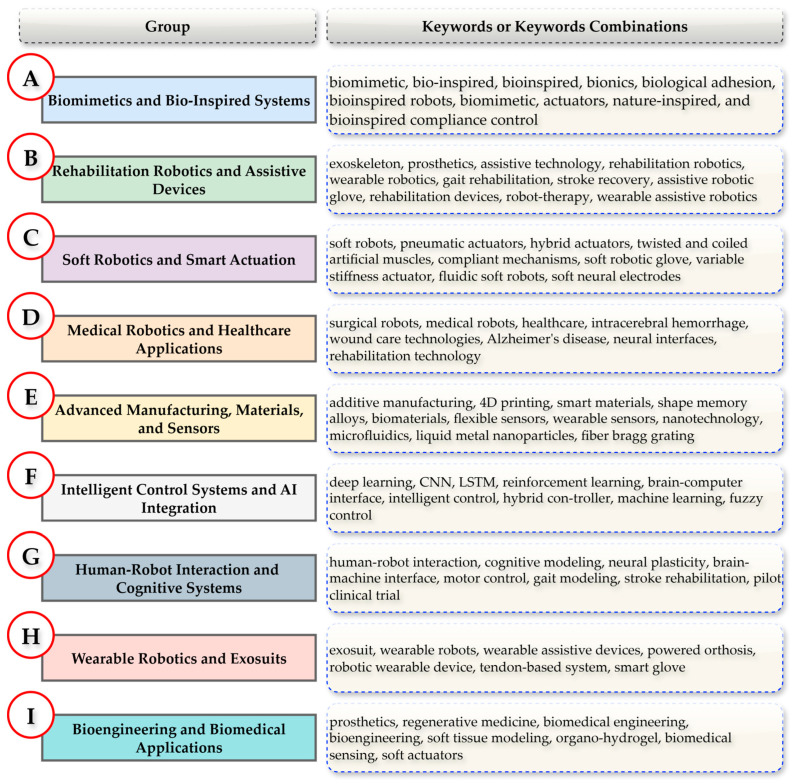
Source group classification based on author keywords or keyword combinations.

To classify the studies by application domain, the authors organized them into nine distinct groups (Group A–I) based on keywords or keyword combinations. As illustrated in Figure 4, the sources were assigned to their respective application domains according to the identified keywords or keyword combinations. The methodology employed for this classification process is detailed in Algorithm 1, which outlines the use of predefined keywords to categorize the sources systematically. This keyword-based approach facilitates the grouping of studies within thematically related domains.
**Algorithm 1: Categorization Based on Keywords or Keyword Combinations**1: Load dataset D ← CSV2: **For** each record d_i_ ∈ D **do**3:    **If** d_i_.k = ∅ **then**4:       d_i_.k ← ""5:    **End**
**if**6:    d_i_.k ← lowercase(d_i_.k)7: **End**
**for**8: Define category-keyword map: C = {c_1_ ↦ K_1_,…, c_n_ ↦ K_n_}9: **For** each d_i_ ∈ D **do**10:    S_i_ ← ∅11:    **For** each (cⱼ, Kⱼ) ∈ C
**do**12:      If ∃k ∈ Kⱼ such that k ∈ d_i_.k **then**13:        S_i_ ← S_i_ ∪ {cⱼ}14:      **End**
**if**15:    **End**
**for**16:    d_i_.c ← S_i_17: **End**
**for**

## 3. Comparative Analysis of Related Works

Section 3.1, Section 3.2, Section 3.3, Section 3.4, Section 3.5, Section 3.6, Section 3.7, Section 3.8, Section 3.9, Section 3.10, Section 3.11, Section 3.12, Section 3.13 and Section 3.14 examine previous studies’ methodologies, conclusions, and technological advancements on biomimetic sensing and robots in healthcare and rehabilitation. The authors emphasize the study’s importance by placing it in the larger context of bio-inspired sensing and robotics for rehabilitation science, classifying each application by domain using abstract and main text information, and pointing out emerging trends, recurrent themes, and research gaps.

### 3.1. Biomimetic Movements, Sensing, and Tactile Interfaces

Replicating human touch and movement has been the focus of recent research in bio-inspired technology for robots and prosthetics. For instance, Verma et al. [11] introduced a high-performance CMOS-based tactile sensor array for stress detection and artificial skin. Ortega et al. [12] created a robotic finger that imitates human movements using an easy-to-make, straightforward design. These studies aimed to enhance the functioning and integration of bio-inspired approaches in assistive technology, robotics, and rehabilitation.

### 3.2. Bio-Inspired Neurological and Metabolic Therapies

The creation of novel neuromodulation methods for neurological conditions has been fueled by bio-inspired engineering. For instance, Seyedbarhagh et al. [13] showed how an FPGA-based deep brain stimulator with CORDIC can model neuron–glial interactions and repair intracellular calcium wave abnormalities in Alzheimer’s disease (AD). Furthermore, a soft robotic device that uses granular jamming to non-invasively reduce Parkinson’s disease (PD) tremors by 84.6% was introduced by Seshadri [14]. Moreover, Ramadi et al. [15] suggested FLASH, an edible capsule that improves gut–brain axis signaling and provides a minimally invasive treatment for neuropsychiatric and metabolic disorders. The design was inspired by the “Thorny devil” lizard.

### 3.3. Prosthetics and Rehabilitation Systems

A biomimetic ankle-foot prosthesis that can replicate natural gait and improve postural control via neural-inspired PID control was introduced in prosthetic development by Mishra et al. [16]. Building on this, Li et al. [17] created a foot made of carbon fiber with curved surfaces to enable human-like, energy-efficient movement. Picolli et al. [18] improved prosthesis actuation by combining a cable-driven system with grasped, elastic components to reduce energy spikes and replicate muscle action. The simulations showed a reduction of 60% in the force peak and 40% in the power peak.

### 3.4. Soft Robotics and Compliant Design

Soft robotics leverages compliant materials and bio-inspired designs to enable safe, adaptable interaction in various applications, from rehabilitation to minimally invasive medical sensing. Soft robotics is essential for simulating organic compliance and mobility, with new developments including structures with adjustable rigidity inspired by fish scales presented by Milad et al. [19]. In surgical training, a peristaltic simulator using pneumatic artificial muscles was introduced to simulate organic movement by Peerlinck et al. [20], while robotic fish joints achieved up to 89% energy efficiency by controlling timing and stiffness, proposed by Chen et al. [21]. In the medical field, teleoperated oropharyngeal swabs have improved safety during remote COVID-19 diagnoses by employing a bio-inspired soft robotic hand [22]. Other significant advances include the modular Soft pneumatic network (Pneu-Net) actuator mimicking an elephant’s trunk for finger rehabilitation, offering a reproducible and energy-efficient approach [23]. Hydraulically soft actuators (HSAs) like the Bio-SHARPE further reduce power and weight requirements [24]. Soft exoskeletons, including a high-DOF glove and a lightweight wrist device, show promise in restoring hand function and dexterity in stroke survivors [25,26]. Compliant control strategies such as variable stiffness actuation (VSA) [27,28] and EMG-driven variable stiffness modulation [28] enhance safety and performance in wearable devices. Nature-inspired soft robots, like caterpillar-like cardiomyocyte-driven robots [29] and kirigami snake robots [30], expand soft robotics into advanced biomedical applications. Finally, integrated soft robotic systems like the TENG-SPA demonstrate promising applications in digital rehabilitation by accurately assessing spasticity levels in patients [31]. These advances demonstrated the transformative potential of soft, compliant designs in human–robot interaction, medical devices, and bio-inspired adaptive systems.

### 3.5. Human Motion Control and Assistance

A gait controller that uses the hip–ankle torque distribution for human motion analysis and control was proposed by Amini et al. [32] and verified in OpenSim to guarantee the stability of a natural walk. A different study by Olikkal et al. [33] demonstrated enhanced human–robot interaction through biomechanical modeling by achieving 95.7% gesture recognition accuracy while controlling a robotic hand with an RGB camera and dimensionality reduction. When multimodal sensing was investigated by Zou et al. [34], the estimation error in exoskeleton control was significantly decreased by combining surface electromyography (sEMG) and ultrasound.

### 3.6. Bio-Inspired Imaging and Surgical Technologies

Liu et al. [35] proposed a bio-inspired multimodal 3D endoscope that improves cancer surgery by utilizing mantis shrimp’s and humans’ superior vision. Compared to current endoscopic systems that sacrifice workflow and lighting, it allows for the simultaneous real-time imaging of three-dimensional stereoscopic, color, and near-infrared (NIR) fluorescence pictures under standard surgical lighting. The endoscope provides surgeons with real-time input without interfering with their workflow by utilizing a multiband sensor, an optical relay system, and a broadband binocular optical system. Its capacity to precisely detect tumor tissue and lymph nodes was confirmed by experiments, indicating potential for broader application in image-guided and robotic surgery.

### 3.7. Convergence of Portable Biosensors and Flexible Electronics

Flexible electronics and portable biosensors are combining to produce more individualized, accessible, and integrated healthcare solutions, according to recent studies [36,37,38]. Electronic systems and biomedical applications are becoming more compatible thanks to advancements in dental technology, wearable technology, and point-of-care (POC) diagnostics. For instance, the Cu-EGaIn-based stretchable e-skin, which was first presented by Guo et al. [36], offered low manufacturing costs by commercial laser printing and remarkable mechanical flexibility and conductivity. Because of its compatibility with CT imaging, it can be used for surgical navigation and human–machine interaction. In the meantime, Kou et al. [37] presented an enzymes@MOFs-based paper biosensor that can be integrated into a smartphone and perform real-time biochemical analysis with small sample volumes. This method improved diagnostic accessibility in low-resource environments using metal-organic framework (MOF) nanoreactors for enzymatic sensing. Hassantash et al. [38] investigated regenerative dentistry utilizing digital tools to enhance clinical results. These innovations demonstrate how biomimetics have revolutionized contemporary diagnosis.

### 3.8. Advances in Biomimetic Rehabilitation Systems

Suppiah et al. [39] presented BIOFIS’s LSTM-based architecture, which enhances the classification of EEG and EMG signals by identifying temporal and inter-sensor correlations. As a result, individuals with severe disabilities can use the system more easily because it improves accuracy even with weak signals. A four-link robotic end-effector with biomimetic muscle actuators and fuzzy logic control adapted to a user’s biomechanics was discussed by Goyal et al. [40], allowing for safer and more accurate rehabilitation. Strong scalability is indicated by its success in subjects who are not affected.

Lv et al. [41] presented a hand exoskeleton for pediatric use. It utilized a predictive model to modify its assistance based on EMG-derived grip strength to promote voluntary effort and motor learning. Because of its real-time adaptation to various gait patterns, the FATP-N model discussed in [42] is perfect for intelligent lower-limb orthotics and prosthetics. It used a single motion sensor to assess foot angles. Genetic algorithms were used by Liang et al. [43] to optimize biomimetic joint design in hand exoskeletons, increasing performance and realism. This technique has the potential to improve assistive technology for many joints.

Yang et al. [44] presented a soft pneumatic actuator with a caterpillar-inspired design. A trilayer-knit textile framework achieved dual-stiffness, high power density, and rapid response features that hold promise for soft robotics and wearable medical equipment. Minimally invasive surgery was supported by the Shurui^®^ single-port robotic system in the study by Hu et al. [45], which has serpentine manipulators and demonstrates efficacy and safety in gynecological procedures. Bio-inspired 3D-printed surgical forceps with improved clamping forces were used by Sun et al. [46] to guarantee sterile, safer surgical tools.

Scalable, flexible support for exoskeletons was provided by a control architecture in the study by Delgado-Oleas et al. [47] that was inspired by human–motor systems and was effectively applied in a gait rehabilitation platform. In the meantime, Varghese et al. [48] outlined a shoulder exosuit sensing system that used motion capture and neural networks to precisely record joint angles, setting the stage for future actuator integration.

When taken as a whole, these studies highlight developments in wearable technology, medical robotics, and biomimetics that provide adaptable and patient-centered solutions.

Table 1 provides a comprehensive overview of notable studies contributing to biomimetic and rehabilitation systems. It categorizes the studies based on the underlying technology, such as bio signals, robotic end-effectors, soft pneumatic actuators, and neuromuscular sensing. It concisely outlines each study’s key innovation discussed under Section 3.8. The categorization was made under the abstract, keywords, and keyword combinations.

### 3.9. Innovations in Biomimetic and Wearable Medical Systems

Technological developments in biomimetic robots and sensing have greatly improved medical diagnostics and rehabilitation. In addition to bio-inspired systems, rehabilitation robots, assistive devices, and intelligent medical platforms, researchers have investigated various topics [31,49,50,51,52,53,54,55]. For instance, a unique adaptive control approach for exoskeletons was developed by Chairez et al. [49]. It uses a Super-Twisting differentiator to improve real-time velocity prediction in unexpected situations and dynamically modifies Proportional-Derivative (PD) gains via a Lyapunov-based law. To help stroke patients’ in-bed rehabilitation, a magnetic pantographic exoskeleton (Mag-PGE) was created by Que et al. [50] with an emphasis on biomechanical compatibility to facilitate ankle movements. Its magnetic coupling and integrated sensors provide precise motion control and feedback, outperforming conventional actuation techniques.

Additionally, Wei et al. [51] used finite element modeling of the human hand to examine tendon routing patterns, highlighting the significance of biomechanical insights for exoskeleton design, namely the lateral bands and extensor hood. Gao et al. [52] presented a soft robotic knee prosthesis with an origami-inspired shape that reflects flexible, user-centered design concepts while allowing for natural motion and stress absorption.

Combining tactile sensors and adaptive actuators created an adhesive multi-functional ionic skin (MiS) by Lin et al. [53] for multimodal sensing and wound healing, allowing for closed-loop control. Chen et al. [54] went one step further to provide comfort and precise tracking for post-stroke rehabilitation. They created a tendon-driven, biomimetic hand exoskeleton customized to finger biomechanics. Finally, these efforts were furthered by Li et al. [31] and Egorov et al. [55] through intelligent control and structural advances. While the latter unveiled the TENG-SPA, a triboelectric soft actuator with AI-enhanced spasticity prediction, demonstrating the potential of AI-integrated adaptive rehabilitation, the former suggested gait-optimized, parameterized exoskeleton designs.

Table 2 summarizes the notable contributions in adaptive, tendon-driven, and customizable exoskeleton technologies for rehabilitation, highlighting innovative design strategies and control methods across recent studies discussed in Section 3.9. The categorization was made under the abstract, keywords, and keyword combinations.

### 3.10. Bio-Inspired Soft Robotics and Smart Actuation

Numerous researchers in biomimetics, bio-inspired systems, soft robotics, and smart actuation have created creative ways to enhance assistive and rehabilitation technologies [23,24,25,26,28]. For example, a modular soft pneumatic actuator was created by Elchrif et al. [23] and comprises four interconnected Pneu-Net modules. This actuator achieves 46% greater energy usage thanks to reinforcing fibers, and it offers multiple degrees of freedom (DoF), improved mechanical compliance, and improved reproducibility. It was created to support finger rehabilitation and was modeled by the curling motion of an elephant’s trunk. The Bio-SHARPE concept, which has a high aspect-ratio soft pumping element that can increase input volume by 8.6 times, was also presented by Davies et al. [24]. This development overcomes the drawbacks of conventional soft actuation systems. It makes smaller, more effective pumping units possible, as seen in applications such as an artificial heart ventricle and a soft robotic squid. Inspired by the human musculoskeletal system, Kim et al. [25] developed a high-DOF soft robotic glove for neuromuscular rehabilitation. It demonstrated a 248% increase in thumb workspace, enhanced object manipulation, and decreased pinching pressures in stroke survivors. Furthermore, Yang et al. [28] created a biomimetic system that combines EMG-based control and variable stiffness actuation for elbow joint rehabilitation. It evaluates joint stiffness and supports a variety of activity intensities while promoting limb coordination through muscle synergy analysis and impedance control. Last, Greco et al. [26] created a lightweight exoskeleton for wrist rehabilitation driven by coiled and twisted artificial muscles (TCAMs). It is lightweight (0.135 kg), enables passive and active-resisted training, and allows multidirectional wrist motions. It is also appropriate for long-term home-based therapy. Table 3 summarizes notable contributions in bio-inspired soft robotics and intelligent actuators, highlighting innovations in soft pneumatic systems, wearable rehabilitation devices, and adaptive actuation technologies discussed in Section 3.10. The categorization was made under the abstract, keywords, and keyword combinations.

### 3.11. Bio-Inspired and Biomimetic Systems for Limb Rehabilitation

According to research by [56,57,58], using bio-inspired, biomimetic, and robotic technologies can improve the biomechanics and functionality of assistive and rehabilitative devices. These developments aim to enhance human-like mobility and performance by focusing on particular mechanical support regions, such as the wrist, hand, or hip. For people with limb loss or disabilities, recent developments in rehabilitation robotics and prosthetic systems are intended to increase upper-limb movement, hand dexterity, and walking efficiency. By lowering early-stance hip extension torque, motorized hip exoskeletons, for example, dramatically reduce net residual hip energy when walking, according to Ishmael et al. [56]. The Walking economy increased due to the net residual hip energy dropping from 0.05 ± 0.04 J/kg without assistance to −0.01 ± 0.05 J/kg with the exoskeleton (*p* = 0.026).

Additionally, advancements in soft robotics have produced a very adaptable rehabilitation glove according to Zheng et al. [57]. A wide range of motion is made possible by this glove’s multimodal, deformable soft fingers, which are powered by numerous soft actuators and driven by a single Degree of Freedom (DOF). Simulations and experiments demonstrate strong potential for assistive and rehabilitative applications. Furthermore, the Hannes hand prosthesis presented by Boccardo et al. [58] incorporated a biomimetic wrist prosthesis with active flexion-extension and pronation-supination. This 2-DOF wrist indicates promising improvements for upper-limb prosthetics since it increases dexterity and closely resembles the range of motion of the native wrist in healthy patients.

For wearable rehabilitative devices, recent bio-inspired device improvements also address biomechanics, comfort, and adaptive design [59,60,61]. Using a bio-inspired tendon-driven mechanism, Su et al. [59] demonstrated a soft wearable skin brace that allows forearm pronation/supination for neurological conditions, making it simple to put on and take off while maintaining range of motion. Sabau et al. [60] used AI to automate and optimize socket design to address user comfort and design time in transfemoral sockets. Gait simulations confirmed improved walking performance and reduced physical testing. An innovative bionic leg orthosis (SBLO), an improvement on conventional KAFOs, was presented by Payra et al. [61] in the meantime. It synchronizes knee motion with the stages of gait. According to biomechanical studies, SBLOs assist polio survivors in regaining a nearly normal stride, which lowers energy expenses and enhances general function.

Table 4 summarizes key innovations in bio-inspired and biomimetic technologies for limb rehabilitation, highlighting advancements in upper-limb prosthetics, soft exoskeletons, and intelligent bracing systems to improve mobility, comfort, and functional performance. The categorization was made under the abstract, keywords, and keyword combinations.

### 3.12. Bio-Inspired Systems in Rehabilitation Robotics

The papers in [62,63] showcase recent advancements in bio-inspired wearable robotics for rehabilitation and assistance. Abdelhafiz et al. [62] developed a novel soft hand exoskeleton that emulates the human musculoskeletal system, featuring a redesigned exotendon routing system. Rearranging the tendons in a single-motor-driven system effectively avoids finger joint hyperextension and better simulates intrinsic muscle functions. Experiments with nine healthy volunteers demonstrated that this design enables natural finger movements and reduces strain at the DIP and MCP joints. It also showed promise for treating spasticity in a rigid prosthetic finger model. Meanwhile, Natali et al. [63] highlighted the XoSoft EU project’s development of a modular, soft exoskeleton for lower-limb support. The quasi-passive actuation system dynamically supports walking by combining an elastic belt with active mechanical components. Testing on a healthy subject revealed the device could deliver up to 150.8% of peak ankle power, underscoring the potential of biomimetic wearable robotics for enhanced mobility and rehabilitation.

### 3.13. Enhancing Mobility and Rehabilitation with Exosuits

The expanding wearable robotics and exosuits field has significantly improved mobility, rehabilitation, and daily life for various populations, notably the elderly and injured. Research demonstrates that biomimetic, bio-inspired, and modular designs can improve exosuit performance and user acceptance, with crucial implications for human–robot interaction and rehabilitation studies [62,63,64,65,66]. For instance, Abdelhafiz et al. [62] focused on a bio-inspired exotendon hand exoskeleton that improves finger trajectories and adapts to spasticity-like conditions. Natali et al. [63] showcased a modular exoskeleton using pneumatic quasi-passive actuation, providing substantial hip (26.6%) and ankle (12.6%) support. Furthermore, Natali et al. [64] showed that the XoSoft exosuit’s hips-plus-ankles (HAA) configuration led to a 47% reduction in compensatory muscle activity and an 8% decrease in metabolic cost, improving muscular synergy. Bai et al. [65] introduced a bionic hydraulic actuator that replicates muscle function, improving power density and lifespan, surpassing traditional electric actuators. Firouzi et al. [66] demonstrated the BATEX exosuit’s energy-efficient performance using compliant actuators, achieving a 9.4% reduction in metabolic cost.

### 3.14. Bio-Inspired Soft Robotics for Rehabilitation and Assistive Wearable Technologies

The recent advances in soft robotics have focused on developing bio-inspired, compliant, and safe actuators for biomedical applications, rehabilitation, and wearable robotics. Several researchers have researched soft robotics and its medical-based uses in [23,24,25,26,27,28,29,30,31,67,68]. New modular-based Pneu-Net actuators (Elchrif et al. [23]) and bio-inspired soft pumping elements (Davies et al. [24]) have been developed to enhance flexibility, modularity, and energy efficiency in assistive devices and soft robots. High-degree-of-freedom soft robotic gloves (Kim et al. [25]) and lightweight wearable exoskeletons (Greco et al. [26]) have been designed to improve grasping ability and assist wrist movements in stroke survivors and patients with neuromuscular diseases. Novel bio-inspired variable stiffness control schemes (Zhu et al. [27]) and variable stiffness modulation strategies (Yang et al. [28]) have also emerged to promote bilateral motor skills and safe interaction. In parallel, bio-inspired soft robots, including crawling cardiomyocyte-driven devices (Sun et al. [29]) and kirigami-based sensor deployment robots (Li et al. [30]), are proposed for real-time monitoring and adaptable locomotion in biomedical scenarios. Finally, a high-force-output triboelectric soft pneumatic actuator (Li et al. [31]) inspired by lobster tails shows promise for objective spasticity assessment, and electro-pneumatic wearable soft robotic gloves (Quach et al. [68]) are in early stages of testing as low-cost hand-assistive devices. Tran et al. [67] introduced a voice-controlled soft robotic glove to aid hand function in spinal injury patients, featuring a novel fabrication method.

Cheng et al. [69] introduced two data-driven control methods for powered prosthetic legs to ensure smooth transitions between walking and stair climbing, focusing on biomimetic performance and seamless kinematics. One method continuously interpolates between activity models, while the other uses optimized switching with smoothing. Experiments with two high-functioning transfemoral amputees showed that the continuous approach achieves more natural transitions regardless of which leg initiates movement, highlighting the potential for improved prosthetic functionality. Gutierrez et al. [70] presented a bio-inspired, fluid-driven exosuit system for assisting knee-bending without external energy sources. Inspired by ice-plant hydraulics, the design uses an event-based energy cycle (EBEC) concept to synchronize energy storage and release with the user’s motion. The EBEC skin prototype demonstrates an innovative “sense-acting” system and suggests future applications for fluid-driven soft machines.

Studies in [26,28,47,56,71,72,73,74,75,76,77,78,79,80,81,82,83,84,85,86,87,88,89,90,91,92,93] engaged in a research study of rehabilitation robotics and assistive devices, biomimetics, and biologically inspired systems. Between these studies [48,56,57,58,69,94] discussed bioengineering and biomedical applications. Moreover, studies in [31,39,40,42,43,48,58,69,95,96,97] discussed the topic of intelligent control systems and AI integration in rehabilitation robotics and biomimetics. Studies in [26,48,69,70] discussed wearable robotics and exosuits in biomimetics-based rehabilitation robotics.

The tendency toward bio-inspired and compliant systems to support human mobility and function recovery is highlighted by recent developments in rehabilitation robots. A study by Shao et al. [71] presented an electronic skin with color-changing, conductive properties for biomedical rehabilitation, showing real-time response in robotic knuckle rehabilitation applications. Several novel devices have been developed by Wang et al. [72], including a cable-driven ankle rehabilitation system that exhibits flexibility and compliance by simulating antagonistic muscles. TiRobot and other navigation-assisted surgical robotics increase the accuracy of ACL reconstruction tunnel placement compared to conventional techniques [73]. Although obstacles are still for even more lightweight designs, soft robotic hand exoskeletons with flexible materials allow for personalized and comfortable movement restoration [74]. Bionic soft exoskeletons for respiration mimic natural breathing to provide extracorporeal respiratory support and achieve real-time adaptation in patients with respiratory challenges [75]. Using biomechanical knowledge and EMG analysis to lower muscular activation, self-balancing exoskeletons such as AutoLEE-II help with various crutch-free activities [76,78]. Elbow exoskeletons use EMG and motion sensors with Assist-as-Needed algorithms to customize support and monitor real-time progress [77]. A new “Orbit Energy” metric that directs recovery torque controls has been used to enhance balance recovery techniques for lower-limb exoskeletons [79]. Improved safety in colonoscopy applications is provided by inchworm-inspired devices on vine-like robots [80]. Dragonfly tail-inspired tendon-based bellows actuators offer exceptional tensile strength for demanding exoskeleton applications [81]. To provide post-stroke patients with an efficient and personalized trajectory, index-finger rehabilitation has been handled with a six-bar mechanism-based exoskeleton that has been refined using sophisticated algorithms [82]. Together, these advancements highlight the potential of biomimetics and flexible control schemes in the upcoming generation of assistive and rehabilitation technology.

To improve dexterity and biomimetic performance for everyday tasks, a wearable hand exoskeleton with symmetric conjugate surface joints has been created to replicate natural finger gripping [83]. Segmented composite proprioceptive bending actuators (SCPBAs), which measure stiffness and extension angles to improve aid in daily tasks, are pneumatic bio-inspired devices introduced for hand rehabilitation [84]. In contrast to conventional devices, the DE-AFO is a soft, silent, biomimetic ankle-foot orthosis that uses electro-active polymer muscles to enhance ankle motion and comfort in children with cerebral palsy [85]. For finger rehabilitation, bio-inspired segmented hybrid bending actuators (SHBAs) offer more natural motion; validated static and finite-element models demonstrated their better performance over continuous constructions [86]. Fingertip force and stiffness are improved for everyday living aid by a flexible, bio-inspired finger exoskeleton built on a two-layered nonparallel spring structure [87]. Pneumatic muscles power a new hand rehabilitation tool that simultaneously passively mobilizes the wrist and finger joints while adjusting to the patient’s pain threshold [88]. Objective measures for clinical applications are highlighted in a review of gait analysis system technologies and a novel calibration test that uses a biomimetic rig [89]. A soft robotic hand exoskeleton with tendon tension sensors, high-consistency silicone, and admittance control for better grasping has been designed by Tran et al. [90] to rehabilitate cervical spinal cord injuries. Improved motor learning and functional outcomes in post-stroke rehabilitation were demonstrated by Asín-Prieto et al. [91] using a novel robotic ankle exoskeleton that uses video-game input and tacit learning control. For stroke patients recovering their gait, VIC-SEATS, a variable impedance compliant actuator for knee joint rehabilitation, provides optimal stiffness, safety, and force control [92]. Last but not least, simulations have confirmed the efficacy of a robotic elbow exoskeleton for limb rehabilitation that uses bio-inspired control techniques and pneumatic artificial muscles (PAMs) [93].

Recent developments in bio-inspired assistive technology and rehabilitation robotics have shown great promise for enhancing mobility and functionality in people with physical disabilities. Lee et al. [94] developed Exo-Abs, a robotic system that assists respiratory functions via synchronized abdominal compression. A powered hip exoskeleton decreased net residual hip energy and extension torque in patients with above-knee amputations, confirming previous findings of better walking economy [56]. Anthropomorphic wrist prostheses with active flexion-extension and pronation-supination capabilities improve dexterity in upper limb prostheses [58]. At the same time, flexible soft glove designs with multimodal deformable fingers provide flexibility and variability for rehabilitation tasks [57]. Exoskeletons using crank-slider series elastic actuators provide accurate, compliant torque control for enhanced human–robot interaction [96]. Hybrid brain–muscle–machine interfaces and variable stiffness modulation techniques highlight the possibility of involving the entire neurological system and modifying robotic compliance to facilitate motor relearning in stroke rehabilitation [28,94]. Furthermore, new control strategies like the FATP-N foot trajectory prediction network [42], soft exoskeletons with twisted-coiled artificial muscles [26], and genetic algorithms for biomimetic joint optimization [43] demonstrate the increasing complexity of soft actuation and wearable robotics. By linking top-down and bottom-up bio-inspired methods, interdisciplinary, bio-inspired designs like fluid-driven event-based energy cycle concepts open the door for assistive devices that are both intuitive and energy-efficient [70]. Prasanna et al. [97] proposed a vibrotactile feedback system to help amputees sense uneven terrain.

The development of exosuits represents a revolutionary advancement in rehabilitation and mobility. These wearable technologies enable people to restore their independence and quality of life by enhancing natural movement and offering tailored support. Exosuits have the potential to completely transform physical rehabilitation, injury prevention, and workplace ergonomics as long as research and technology continue to progress. In the end, exosuits signify a future in which mobility is a bridge to opportunity rather than a barrier, and they also symbolize the blending of engineering and human physiology.

## 4. Discussion

In recent years, there has been a steady increase in the general trajectory of research in biomimetic robotics and sensing for healthcare applications and rehabilitation, indicating a greater understanding of its revolutionary potential. The number of research studies increased dramatically from 11 to 28 between 2020 and 2023, indicating increased interest and potential enhancements in resource allocation and field-wide collaboration. Significant advancements, such as creating more sophisticated and efficient biomimetic devices, are probably the result of such a surge. Additionally, 20 research studies were still conducted in 2024, demonstrating that this high activity level has continued past the 2023 peak and is not a fad. These encouraging patterns indicate a growing corpus of research emphasizing the significance of biomimetic strategies for assistive healthcare and rehabilitation devices. The expanding body of research may reveal the wider attractiveness of bio-inspired design in addressing complex patient care issues like improving sensory feedback, boosting patient engagement in treatment, and improving mobility. Transforming this study into clinical practice will require sustained funding and interdisciplinary cooperation, guaranteeing that the advancements of the past few years result in observable, practical advantages for both patients and caregivers.

**Figure 5:**
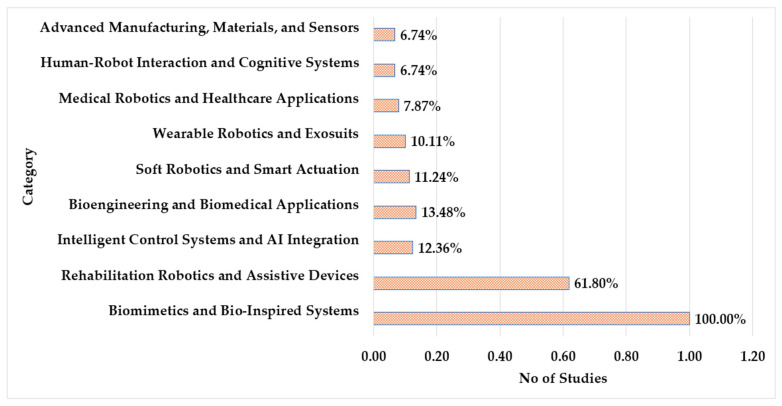
Distribution of the sources by technological categories. Categories include biomimetics and bio-inspired systems, rehabilitation robotics and assistive devices, intelligent control and AI integration, bioengineering and biomedical applications, soft robotics and smart actuation, wearable robotics and exosuits, medical robotics and healthcare applications, and other emerging interdisciplinary areas. Percentages reflect the proportion of studies aligned with each domain based on keywords and keyword combinations.

The frequency of domain usages in robotics and related disciplines across different categories is shown in Figure 5. With 89 occurrences, biomimetics and bio-inspired systems are in the lead, demonstrating their crucial importance in contemporary research and applications. The rising emphasis on healthcare and human mobility solutions is reflected in the 55 instances (61.8%) of rehabilitation robotics and assistive devices that follow. The integration of intelligent algorithms in robotics is highlighted by the 11 appearances (12.36%) of intelligent control systems and AI integration. Innovation in responsive and flexible systems is emphasized in soft robotics with smart actuation (10; 11.24%) and bioengineering and biomedical applications (12; 13.48%). Medical robotics and healthcare applications (7; 7.87%) represent particular applications in healthcare settings, while wearable robotics and exosuits (9; 10.11%) indicate developments in enhancing human capabilities. Emerging multidisciplinary interests are shown by the six appearances (6.74%) of advanced manufacturing, cognitive systems, and human–robot interaction.

Table 5 shows the summary of the review study based on the keywords or keyword combinations available in the main content of the study. The 2020–early 2025 review study groups publications into Groups A–I, emphasizing the interconnection and changing research focuses. With significant overlaps with Groups B, E, and G, Group A had the most references in 2020, indicating foundational explorations. With pieces like [48,55,60,63,91,92,98], Group B strongly overlapped with Group A, suggesting their interests were similar. Group A led the 2021 landscape, while Groups B and G gained importance, while Groups E and H experienced targeted action. Group A was again in the center by 2022, with growth in Groups B and D following suit, indicating more sophisticated research areas.

Groups C and G continued to be active, but less so. Research output exploded in 2023, with Group A having the most references (see Table 5), indicating a more thorough thematic investigation. Significant representation was shown by Groups B and F, and Group I’s participation increased as well. Group B (see Table 5) was the most active during the consolidation phase that began in 2024, while Groups G, H, and I concentrated on recurring themes. Data from January through May of 2025 indicate a slower start, with Groups H and I, while Group B ([31,78,80]) maintained concentration. This progression highlights research themes that are both dynamic and interrelated.

**Figure 6:**
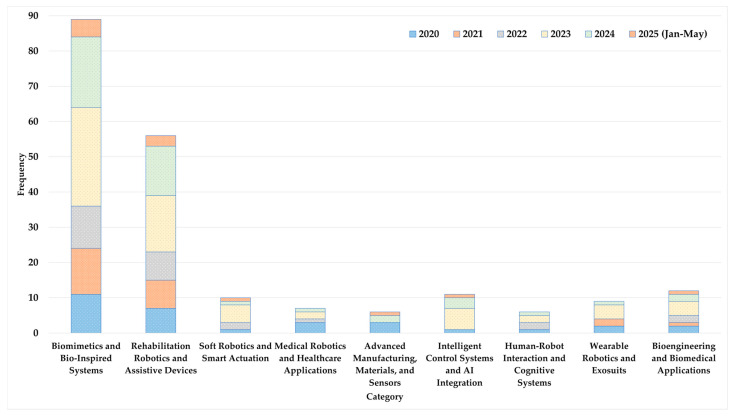
Year-wise domain category addressed by the related studies.

As shown in Figure 6, research in bioengineering and robotics exhibits noteworthy trends in several domains between 2020 and early 2025. Strong interest in nature-inspired solutions is demonstrated by the consistent leadership of biomimetics and bio-inspired systems, which peaked in 2023 with 28 studies. The steady growth of rehabilitation robotics and assistive devices focuses on enhancing mobility and aiding technology. There is growing interest in flexible robotics, as seen by the moderate but erratic attention given to soft robotics and intelligent actuators. With a focus on continuous healthcare innovation, medical robotics and healthcare applications continue to be active, although at a slower pace. The relevance of advanced manufacturing, materials, sensors, intelligent control systems, and AI integration is increasing. In particular, AI saw a sharp increase in 2023, demonstrating the expanding use of intelligent algorithms. Wearable robotics, bioengineering, and human–robot interaction all make steady but minor contributions, highlighting biomedical and human-centered methods. The evidence points to a vibrant research environment centered on biomimicry, rehabilitation, integrating AI, and improving human–robot cooperation.

**Figure 7:**
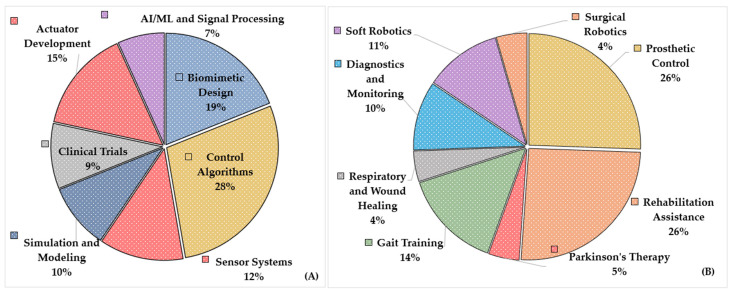
The distribution of the studies is based on (**A**) clustering by methodologies and (**B**) clustering by objectives.

Figure 7A shows the distribution of the studies related to the culmination of methodologies, and Figure 7B shows clustering based on objectives. Control algorithms dominate the methodological landscape with about 28.4% of the 74 papers. The field’s emphasis on improving feedback systems, adaptive control, and real-time regulation to provide stable and responsive robotic behavior is highlighted by this prominence. The remaining 18.9% of studies are in biomimetic design, which reflects continuous attempts to mimic biological structures and functions in the choice of materials and technology. More than a quarter of the research was focused on sensor systems (12.2%) and actuator development (14.9%), which emphasize ongoing innovation in the “muscle and nervous system” analogs of robotic devices. In the meantime, clinical trials, modeling, and simulation account for roughly 9.5% of the total, showing that theoretical validation and translational research are given equal weight. Lastly, the 6.8% share of AI/ML and signal processing indicates an increasing, albeit still infant, interest in using data-driven methods for pattern identification and decision making.

Prosthetic control and rehabilitation aid are tied for the most common purposes (25.6%) when grouped by objectives (90 studies). This parity indicates that almost half of the selected sources’ research community focused on developing devices and interfaces for rehabilitation and assistance that facilitate movement recovery, which indicates priorities and clinical demand. In total, 14.4% is made up of gait training, which represents specialist therapies for ambulation issues. Diagnostics/monitoring (10.0%) and soft robotics (11.1%) highlight the drive for patient-friendly, compliant equipment and ongoing health monitoring. Other specialized fields include surgical robotics, respiratory and wound healing, and Parkinson’s therapy, each accounting for about 4.4%. These smaller groups could indicate highly specialized or emerging subfields ready to expand.

**Figure 8:**
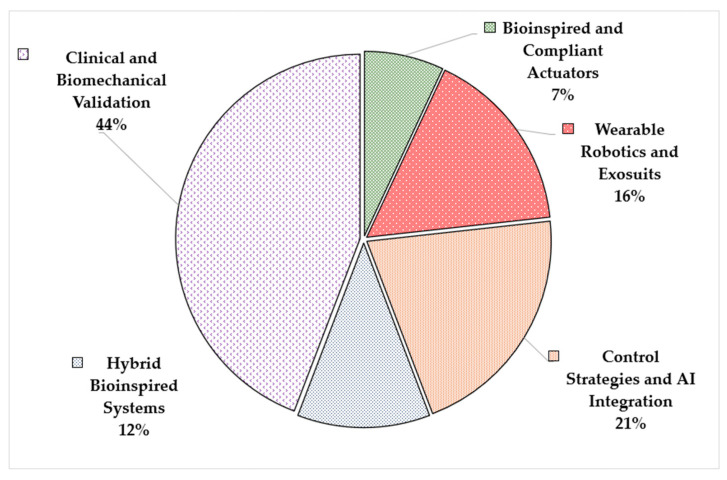
The application and approach involved in the bio-inspired exoskeletons and robotics domain.

As shown in Figure 8, the analysis of 43 studies focusing on “bio-inspired exoskeletons and robotics studies” reveals a precise distribution of research efforts. Clinical and biomechanical validation emerges as the most explored topic, accounting for 44.19% of the studies. This emphasizes real-world applications and patient outcomes, reflecting the field’s growing maturity and importance in healthcare. Control Strategies and AI Integration also receive significant attention (20.93%), likely due to the critical role of adaptive algorithms in ensuring safe, responsive interaction between humans and exoskeletons. Research on Wearable Robotics and Exosuits (16.28%) and Hybrid Bio-inspired Systems (11.63%) highlights ongoing efforts to refine and personalize wearable devices. However, Bio-inspired and Compliant Actuators remain underexplored (6.98%), suggesting an opportunity for further innovation in actuation technologies. The distribution reflects a balanced but application-focused research landscape, prioritizing translational impact while recognizing the need for continued technological advancement.

Each abstract and keywords or keyword combinations were examined to identify the specific AI methodology employed, such as neural networks, fuzzy logic, genetic algorithms, etc., based on both explicit mentions and contextual descriptions of algorithmic frameworks. Table 6 presents an overview of rehabilitation-focused research studies, highlighting the AI methodologies utilized and summarizing the primary contributions discussed in each study.

The prevalence of biomimetics, bio-inspired systems, and rehabilitation robotics in the frequency analysis and the expanding amount of research indicate that nature-inspired solutions are evolving from specialized experiments to become essential components of contemporary healthcare robotics. Intelligent control systems, AI integration, and soft robotics are examples of how biology, engineering, and data science are coming together to provide responsive, flexible, and patient-specific solutions. Though the quick development and clinical testing of bio-inspired exoskeletons and robotics hold out the promise of improving mobility and rehabilitation results, they also bring with them implementation challenges, including the requirement for strong safety regulations, well-defined regulatory processes, and ethical concerns about data privacy in AI-driven systems. Furthermore, the uneven emphasis on actuator development and understudied areas like bio-inspired compliant actuators suggests gaps that need to be filled to realize these devices’ full therapeutic potential, even though the ubiquity of clinical validation initiatives indicates that the field is growing. Additionally, these bio-inspired methods promise more intuitive and effective patient care by utilizing human-like flexibility to develop devices that smoothly integrate with natural movement and offer subtle sensory feedback, essential for engagement and rehabilitation. These technologies’ capacity to handle intricate patient care problems, such as enhancing mobility and providing individualized support, can significantly improve patients’ quality of life while lessening the strain on healthcare systems and caregivers.

In addition to cataloging technological trends, the reviewed studies provide meaningful insights into how biomimetic and bio-inspired systems translate into real-world healthcare settings. For example, high-DOF soft robotic gloves [25], tendon-driven exosuits [54], and pediatric hand exoskeletons [41] have moved beyond laboratory proof-of-concept. They are undergoing clinical testing to support patient-specific motor recovery. Similarly, adaptive control strategies such as EMG-driven impedance modulation [28,40] demonstrate strong potential for personalized rehabilitation programs, particularly for stroke or neurodegenerative disease patients. These developments underscore biomimetic devices’ increasing clinical relevance and ability to integrate with real-time feedback systems, thereby enhancing patient outcomes in naturalistic rehabilitation scenarios.

Significantly, wearable robotic technologies, such as the Mag-PGE [50] and TENG-SPA [31], demonstrate how rehabilitation tools are becoming more portable, lightweight, and responsive to patient needs, essential for adoption in home-based care and telemedicine models. Using low-cost, smartphone-integrated biosensors [37] and paper-based diagnostics also reflects a significant shift toward scalable solutions in low-resource environments, extending the global reach of healthcare innovations.

Future research should not only focus on refining technical components such as compliant actuators and AI-driven control systems but also prioritize deployment strategies. These include co-design with clinicians, rigorous longitudinal trials, and economic assessments to evaluate cost-benefit trade-offs. Furthermore, unresolved challenges such as signal degradation in dynamic environments, power efficiency in wearable systems, and dataset scarcity for AI training demand targeted research efforts. By focusing on these areas, the field can ensure that technological advances meaningfully translate into improved care delivery and enhanced quality of life for patients.

However, several critical challenges must be addressed to fully realize the clinical and practical potential of biomimetic and bio-inspired healthcare robotics. Packaging constraints, power consumption, and the overall hardware footprint represent significant barriers to widespread deployment. The reviewed literature underscores the importance of compact, lightweight designs that integrate multiple sensing and actuation components without compromising performance. Power efficiency is especially vital for mobile or wearable systems, prompting the exploration of strategies such as low-power electronics, duty-cycled operation, and energy harvesting through solar, thermal, or vibrational sources. Modular and scalable hardware designs are also emphasized for greater adaptability across diverse clinical environments. Despite these proposed solutions, detailed considerations of packaging techniques, system robustness, and long-term durability under realistic operating conditions remain relatively scarce. These technical limitations are compounded by broader issues, including reliance on complex and often costly materials and fabrication methods, which can hinder scalability and exacerbate disparities in access to care.

To better align biomimetic innovations with real-world clinical applications and to guide future research, Table 7 summarizes common current limitations and outlines actionable research directions across key domains in this field. These insights reinforce the need for translational frameworks that integrate patient feedback, economic viability, and long-term usability to ensure the success of next-generation biomimetic systems in everyday clinical practice.

Additionally, integrating AI and data-driven systems raises concerns about algorithmic bias, data privacy, and the risk of overdependence on automated decision-making that may fail to account for patient-specific nuances. The technical complexity of these systems also necessitates specialized expertise for operation and maintenance, posing potential barriers in healthcare settings where training resources may be limited. Addressing these multifaceted challenges requires a comprehensive, interdisciplinary approach that fosters collaboration across engineering, biology, clinical practice, ethics, and policy development. Advancing inclusivity, ensuring robust ethical safeguards, and promoting innovation in fundamental technologies and practical deployment strategies will transition these systems from promising research into impactful healthcare solutions.

## 5. Conclusions

This thorough examination of biomimetic sensing and robots and their applications in healthcare highlights the field’s revolutionary potential to improve patient care and rehabilitation. A review of 89 research studies conducted between 2020 and 2025 shows increased scholarly interest in and understanding of how bio-inspired design might improve mobility, independence, and quality of life. Rehabilitative and assistive robots account for 61.8% of the study, which is noteworthy since it shows how important it is to improve human mobility and day-to-day functioning. Integrating AI and intelligent control systems indicates a promising trend toward adaptive, context-aware technologies (12.36%). In comparison, developments in bioengineering (13.48%) and soft robotics (11.24%) indicate interesting advancements in actuation systems and materials. Significant obstacles still exist despite these successes, such as the lack of shared multimodal datasets, restricted sensor–actuator co-design, and unclear translational pathways. A comprehensive strategy is required to address these issues: first, by establishing open, shared data platforms that the entire community can use and contribute to; second, by developing interoperable digital twins and middleware that integrate AI with modular hardware designs; and third, by creating simple pathways that connect clinical testing, research, and real-world applications. When grounded in clinical feasibility and user-centric design, this ecosystem-level approach can bridge the gap between innovation and implementation, ultimately transforming research breakthroughs into scalable, patient-ready technologies.

## Figures and Tables

**Table 1:** Key contributions of the studies to advancing biomimetic and rehabilitation systems.

Technologies	Study	Key Contribution
Bio signals	[39] *	EEG and EMG classification by capturing temporal and inter-sensor correlations, enhancing inclusivity for users with severe disabilities.
Robotic End-Effectors and Exoskeletons	[40] *	a four-link robotic end-effector using fuzzy logic control and biomimetic muscle actuators for precise rehabilitation tailored to the user’s biomechanics.
	[41]	a pediatric hand exoskeleton with predictive modeling to adapt support levels based on EMG-derived grip strength.
	[42] *	proposed FATP-N, a single motion sensor-based foot angle estimator, ideal for lower-limb orthotics and prosthetics.
	[43] *	optimized biomimetic joint structures in hand exoskeletons using genetic algorithms, boosting performance and natural motion.
Soft Pneumatic Actuators and Surgical Robotics	[44]	Presented a dual-stiffness soft pneumatic actuator inspired by caterpillars, promising for wearable devices and soft robotics
	[45]	The Shurui^®^ single-port robotic system with bio-inspired serpentine manipulators in gynecological surgery achieves minimal invasiveness.
	[46]	Introduced bio-inspired, 3D-printed surgical forceps for enhanced clamping forces in open surgery.
Neuromuscular and Motion Sensing	[47]	Proposed a human-inspired, modular control architecture for exoskeletons, proven effective in gait rehabilitation.
	[48] *	Described a shoulder exosuit sensing system using muscle synergies and proprioception, achieving precise joint angle mapping with low error.

* AI methodologies have been discussed.

**Table 2:** Key contributions of studies focusing on adaptive, tendon-driven, and customizable exoskeleton designs for rehabilitation applications.

Technologies	Study	Key Contribution
Adaptive and Magnetic Exoskeletons	[49]	Introduced a PD gain adaptation law for exoskeletons to manage uncertain environments.
	[50]	Developed a magnetic pantographic exoskeleton (Mag-PGE) to improve ankle mobility and stroke rehabilitation through biomechanical compatibility.
Biomechanical and Tendon-Driven Designs	[51]	Used tendon routing analysis in hand models to refine exoskeleton design.
	[52]	Created an origami-inspired soft prosthetic knee with impact absorption.
	[53]	Introduced MiS, an ionic skin for wound healing and multimodal sensing.
Customizable Hand/Leg Exoskeletons	[54]	Presented a tendon-driven exoskeleton tailored to finger biomechanics.
	[31,55]	Bioinspired rehabilitation robotics using optimization and sensing technologies to enhance motion control, spasticity assessment, and therapeutic effectiveness.

**Table 3:** Key contributions of the studies under soft pneumatics and soft robotics.

Technologies	Study	Key Contribution
Soft Pneumatic Modules and Pumps	[23]	Designed a modular soft actuator inspired by an elephant trunk’s curling motion, supporting finger rehabilitation.
	[24]	Developed a high-aspect-ratio pumping element for compact medical pumps, which is shown in applications like soft robotic squid and artificial ventricles.
Soft Robotic Gloves and Elbow Exoskeletons	[25]	Created a high-DOF glove based on musculoskeletal structures for stroke rehabilitation.
	[26]	Built a lightweight wrist exoskeleton using twisted and coiled artificial muscles (TCAMs), enabling multidirectional motion.
	[28]	Developed a variable stiffness elbow system leveraging EMG and impedance control for better limb coordination.

**Table 4:** Key contributions of the studies under bio-inspired and biomimetic systems for limb rehabilitation.

Technologies	Study	Key Contribution
Upper-Limb Prosthetics and Exoskeletons	[56]	A powered hip exoskeleton significantly reduced residual hip energy expenditure, improving walking economy.
	[57]	Developed a flexible, soft-fingered rehabilitation glove for diverse motion needs.
	[58]	Introduced a 2-DOF anthropomorphic wrist prosthesis, enhancing hand prosthetic dexterity.
Soft Bracing and Socket Design	[59]	Proposed a tendon-driven skin-brace to support forearm motion without compromising other joints.
	[60]	Leveraged AI for customized transfemoral socket design, reducing discomfort and testing needs.
	[61]	Introduced the SBLO for dynamic knee support during gait, significantly improving gait performance in post-polio patients.

**Table 5:** Summary of the review study (based on the author’s keywords of the main content).

Year	Group A	Group B	Group C	Group D	Group E	Group F	Group G	Group H	Group I
2020	[29,36,37,46,48,55,60,63,91,92,98]	[48,55,60,63,91,92,98]	[29]	[46,48,55],	[29,36,37]	[48]	[98]	[48,63]	[48,60]
2021	[18,21,22,35,53,54,61,66,70,88,89,90,93]	[53,54,61,70,88,89,90,93]						[66,70]	[61]
2022	[25,34,41,49,51,59,67,68,71,73,94,99]	[41,49,51,59,67,71,73,94]	[25,68]	[67]			[94,99]		[59,94]
2023	[13,14,15,17,19,24,26,27,28,30,39,40,42,43,44,47,56,57,62,64,65,72,77,81,83,86,95,97]	[26,28,42,43,47,56,57,62,64,72,77,81,83,86,95,97]	[24,26,27,28,30]	[44,47]		[39,40,42,43,95,97]	[64,95]	[26,62,64,65]	[24,30,56,57]
2024	[11,12,16,20,23,33,45,50,52,58,69,74,75,76,79,82,84,85,87,96]	[12,50,52,58,69,74,75,76,79,82,84,85,87,96]	[23]	[45]	[11,12]	[58,69,96]	[96]	[69]	[58,69]
2025 (Jan–May)	[31,32,38,78,80]	[31,78,80]	[31]		[38]	[31]			[38]

Group A: Biomimetics and Bio-Inspired Systems, Group B: Rehabilitation Robotics and Assistive Devices, Group C: Soft Robotics and Smart Actuation, Group D: Medical Robotics and Healthcare Applications, Group E: Advanced Manufacturing, Materials, and Sensors, Group F: Intelligent Control Systems and AI Integration, Group G: Human–Robot Interaction and Cognitive Systems, Group H: Wearable Robotics and Exosuits, Group I: Bioengineering and Biomedical Applications.

**Table 6:** Summary of the AI methodologies used by the various researchers.

Study	Summary	AI Methodologies Used
[31]	Develops a triboelectric soft actuator (TENG-SPA) for spastic hand therapy and integrates a CNN to classify spasticity levels.	CNN
[39]	Proposes BIOFIS, a novel classification model using LSTM to fuse EEG/EMG signals.	LSTM
[40]	Develops a wrist rehab robot with biomimetic muscle actuators and a fuzzy-based adaptive controller.	Fuzzy Systems
[42]	Proposes a temporal CNN model (FATP-N) for online prediction of foot angles while walking using shank data.	CNN
[43]	Optimizes biomimetic joint parameters in a hand exoskeleton using genetic algorithms.	Genetic Algorithms
[48]	Develops a sensor framework for a shoulder exosuit using ANN-based multivariate regression to predict joint angles.	ANN
[58]	Introduces a biomimetic wrist prosthesis with active DoFs integrated with the Hannes hand.	NLR classifiers
[69]	Compares CVT and OST models for prosthetic leg control.	CVT and OST
[95]	Designs a CS-SEA actuator for a knee exoskeleton with neuro-fuzzy sliding mode control.	Neuro-Fuzzy Systems
[96]	Presents a hybrid brain–muscle interface (hBMI) for 7-DoF upper-limb exoskeleton control.	Ridge regression and binary classification
[97]	Introduces a vibrotactile feedback system for terrain recognition via foot insoles.	KNN classifier

Note: CNN: Convolutional Neural Network, LSTM: Long Short-Term Memory, ANN: Artificial Neural Network, NLR: Non-Linear Regression, CVT: Continuously Varying Transition, OST: Optimal Switching Transition, KNN: k-Nearest Neighbor.

**Table 7:** Challenges and future research directions by technology domain.

Technology Domain	Current Limitations	Future Research Directions
Soft Actuators	Limited durability, complex fabrication, and power inefficiency.	Develop biodegradable and energy-efficient materials with scalable production methods.
Wearable Exosuits	User discomfort, difficulty in donning/doffing, and limited clinical testing.	Enhance ergonomic design and conduct long-term trials in clinical populations.
AI-Controlled Prosthetics	Insufficient training datasets, algorithmic bias, and latency in response.	Build open-access, diverse datasets and focus on explainable AI for safety.
Flexible Biosensors	Low signal fidelity in dynamic conditions, integration issues.	Improve real-time signal processing and develop hybrid sensing platforms.
Bio-inspired Surgical Tools	High cost, lack of modularity, and limited surgeon training.	Design cost-effective, modular systems and create simulation-based training tools.
